# Recovery of Physical Function in a Patient with Intensive Care Unit-acquired Weakness after Valvular Surgery: A Case Report

**DOI:** 10.1298/ptr.25-E10366

**Published:** 2026-02-12

**Authors:** Kosho OHTA, Hiroshi SAITO, Mami SHIMIZU, Hiroaki TANABE

**Affiliations:** 1 Department of Rehabilitation, Kameda Medical Center, Japan; 2 Department of Rehabilitation, Kameda Rehabilitation Hospital, Japan; 3 Department of Cardiovascular Surgery, Kameda Medical Center, Japan

**Keywords:** ICU-acquired weakness (ICUAW), Functional recovery, Preoperative physical function, EQ-5D

## Abstract

**Objectives:**

This report describes the functional recovery of a patient who developed severe intensive care unit-acquired weakness (ICUAW) following aortic valve replacement (AVR). It focuses on the utility of preoperative physical function assessment and changes in patient-reported health-related quality of life (HRQOL).

**Case Presentation:**

The patient was a woman in her 70s who developed ICUAW after undergoing AVR. Preoperative assessments included the Barthel Index (BI), Short Physical Performance Battery, gait speed, and grip strength to evaluate physical function, and the EuroQol 5 Dimensions 3 Levels (EQ-5D-3L) to assess HRQOL. A progressive rehabilitation program was initiated after the surgery. Postoperatively, we regularly assessed the Medical Research Council sum score (MRCss) and functional performance metrics, and we tracked HRQOL using the EQ-5D-3L over time.

**Discussion:**

The MRCss of the patient was 16 at ICU discharge, indicating severe muscle weakness. Based on preoperative assessments, tailored rehabilitation goals were set, and the patient was transferred to a post-acute rehabilitation facility. The HRQOL trajectory reached its lowest score on postoperative day (POD) 27 (EQ-5D-3L: −0.166) but gradually improved over time. By POD 102, the patient had achieved near-complete functional recovery with an MRCss of 57, BI of 95, gait speed of 1.33 m/s, and EQ-5D-3L of 0.746 and was discharged.

**Conclusions:**

Functional recovery may be possible even in cases of severe ICUAW with marked muscle weakness at ICU discharge. Preoperative physical function assessments may provide valuable insights into recovery potential and guide rehabilitation planning, thereby contributing to an improved quality of care.

## Introduction

Intensive care unit-acquired weakness (ICUAW) is a syndrome characterized by generalized limb muscle weakness in the absence of any apparent cause other than critical illness^1)^. It is a common complication, occurring in approximately 43% of ICU patients^2,3)^. Therefore, the development of ICUAW should always be considered in patients admitted to the ICU. The Medical Research Council sum score (MRCss) is the recommended method for diagnosis^1,4)^. ICUAW is diagnosed when the MRCss is less than 48, whereas severe ICUAW is defined as an MRCss of less than 36^5)^. In addition, health-related quality of life (QOL) assessed by SF-36 has been reported to remain impaired 6 months after hospital discharge^6)^.

According to a clinical review of ICUAW, approximately 28% of the affected patients develop severe long-term sequelae. Recovery may require several weeks to months; in the most severe cases, complete recovery may not be achievable^4)^. Previous reports have shown that the MRCss at ICU discharge typically ranges from 40 to 50 points^7,8)^. However, the detailed timeline and final extent of physical recovery remain insufficiently established^3,4)^.

In the present case, the patient met the diagnostic criteria for ICUAW, with an MRCss of 16 at ICU discharge, which was well below the threshold of 36 used to define severe ICUAW^5)^, making recovery difficult to predict.

Although many studies have focused on preventing ICUAW and the functional decline and recovery following its onset, identifying the pre-ICU physical function of a patient is important for predicting their recovery trajectory. In particular, for patients undergoing elective surgery, preoperative physical function assessment is feasible and may help predict recovery following the development of ICUAW.

This case report describes a patient who developed ICUAW after undergoing aortic valve replacement (AVR) for symptomatic aortic stenosis, focusing on the significance of preoperative evaluation and the subsequent course of functional recovery. Particular attention was given to the notable fact that, despite an extremely low MRCss at ICU discharge, the patient achieved substantial recovery over a prolonged postoperative course.

## Case Presentation

The patient was a woman in her 70s (height: 152 cm; weight: 51 kg; body mass index [BMI]: 22.1) with an active lifestyle, including traveling and participating in chorus activities. She presented with exertional dyspnea, had a history of comorbid chronic obstructive pulmonary disease and hyperthyroidism, and was diagnosed with symptomatic aortic stenosis (Table 1). She was admitted for AVR and left atrial appendage resection. Preoperative assessments indicated good physical function, with a Barthel Index (BI) score of 100, Short Physical Performance Battery (SPPB) score of 12, usual gait speed of 1.14 m/s, and handgrip strength of 20.9 kg. However, her QOL score, as measured by the utility index of the EuroQol 5 Dimensions 3 Levels (EQ-5D-3L), was relatively low (0.416). The EQ-5D-3L utility index ranges from 0 (equivalent to death) to 1 (perfect health), with scores below 0 indicating health states considered worse than death^9)^. Among Japanese women aged 70 years and older, the average EQ-5D-3L score is reported to be 0.808^10)^.

**Table 1.** Preoperative clinical characteristics

Variable	Value
Aortic valve area (cm^2^)	0.75
Mean pressure gradient (mmHg)	45.9
Peak velocity (m/s)	4.32
BNP (pg/mL)	94.5
LVEF (%)	74.0
NYHA class	II
COPD group	A
STS score (risk of mortality, %)	2.393
Clinical Frailty Scale	4

BNP, B-type natriuretic peptide; LVEF, left ventricular ejection fraction; NYHA, New York Heart Association; COPD, chronic obstructive pulmonary disease; STS, The Society of Thoracic Surgeons

Treatment interventions and the rehabilitation course are described in separate sections; their temporal alignment is summarized in Supplemental Table 1.

### Postoperative course and therapeutic interventions

After the surgery, the patient was admitted to the ICU under mechanical ventilation. She developed respiratory failure due to re-expansion pulmonary edema and pneumonia, which necessitated veno-venous extracorporeal membrane oxygenation (VV-ECMO) support and antibiotic therapy. A registered dietitian intervened on postoperative day (POD) 2 to initiate stepwise enteral nutrition. At that time, nutritional risk was identified with a Mini Nutritional Assessment Short-Form (MNA-SF) score of 6 (preoperative score, 13). VV-ECMO support was discontinued on POD 10. During the ICU stay, systemic severity gradually improved with decreasing inflammation, tapering sedation, and discontinuation of vasoactive agents (Supplemental Table 2). The timing and progression of mobilization were determined in parallel with this systemic stabilization. Mechanical ventilation was withdrawn on POD 21, and respiratory support was transitioned to high-flow nasal cannula, followed by transfer from the ICU to the high-care unit (HCU). On POD 24, a peripherally inserted central catheter (PICC) was placed to secure reliable access for daily antibiotics and to enable initiation of total parenteral nutrition (TPN). On POD 27, following a swallowing assessment by a speech-language therapist (SLT), oral intake was deemed feasible, but poor appetite persisted, and the nutrition support team was engaged. As oral intake stabilized, enteral nutrition was discontinued on POD 38. The patient was transferred to a post-acute rehabilitation facility on POD 74 and discharged on POD 102. Body weight reached a minimum of 44.2 kg (BMI 19.1) on POD 69 and was 45.4 kg (BMI 19.7) at discharge.

Key therapies pertinent to mobilization and recovery included vasoactive support (norepinephrine, epinephrine, dobutamine) and sedation/analgesia (propofol, fentanyl) during the ventilated phase, together with prolonged antibiotic therapy (piperacillin–tazobactam, vancomycin) for infection control. Complementary Kampo therapy, supervised by a Kampo medicine specialist, was used as supportive care. Other medications were part of standard ICU management.

### Rehabilitation interventions and clinical course

**Fig. 1.**
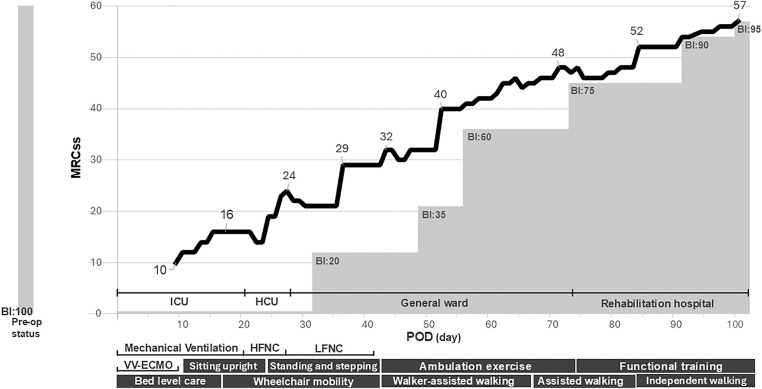
Time course of physical recovery following ICU-acquired weakness. BI, Barthel Index; Pre-op, pre-operation; MRCss, Medical Research Council sum score; ICU, intensive care unit; HCU, high-care unit; POD, postoperative day; HFNC, high-flow nasal cannula; LFNC, low-flow nasal cannula; VV-ECMO, veno-venous extracorporeal membrane oxygenation

**Fig. 2.**
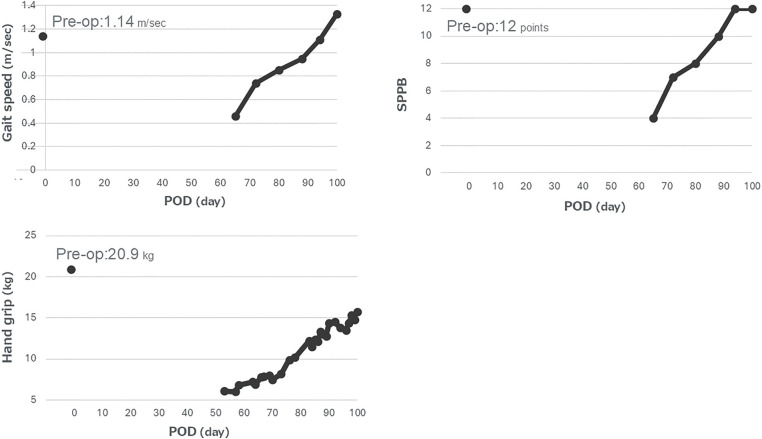
Changes in functional performance metrics during postoperative recovery. Pre-op, pre-operation; POD, postoperative day; SPPB, short physical performance battery

According to the Japanese Circulation Society/the Japanese Association of Cardiac Rehabilitation (JCS/JACR) 2021 Guideline on Rehabilitation in Patients with Cardiovascular Disease^11)^, a progressive rehabilitation program was initiated, including sitting training, marching in place, and walking exercises within the ward. Assisted functional mobility training along with a range-of-motion and strengthening exercises were provided by physical and occupational therapists once or twice daily, 6–7 days per week. Strengthening followed a graded progression: beginning with active-assisted movements and against-gravity tasks, then advancing to progressive resistance training (PRT) with hand-held weights as tolerated^12)^. Intensity was kept at light to moderate effort initially and increased when MRC grades reached ≥3/5. Two to three sets of 10 repetitions were used with adequate rest, and loads were adjusted to maintain a moderate level of perceived exertion. The MRCss, BI, and physical performance were regularly assessed throughout the hospital stay and recorded over time (Figs. 1 and 2). The patient also received encouragement and feedback when appropriate during the rehabilitation process.

In the ICU (POD 0–21), postural drainage and passive range-of-motion were performed during VV-ECMO management; initial mobilization began on POD 10 after VV-ECMO weaning. At that time, the patient exhibited flaccid quadriparesis (MRCss 10; BI 0) requiring full assistance, fulfilling diagnostic criteria for ICUAW. In the HCU (POD 21–27), training focused on transfers and assisted sitting, with supported standing and marching introduced from approximately POD 23. At ICU discharge (POD 21), the MRCss was 16 and the BI was 0; on transfer from the HCU to the general ward (POD 27), the MRCss had slightly improved to 19 while the BI remained 0, and the EQ-5D-3L utility score was −0.166. On the general ward (POD 27–74), gait training began around POD 41 with a walker and total assistance; at that time, the patient could walk 1 m with assistance (MRCss 29; BI 20). Strengthening progressed from active/active-assisted and against-gravity tasks, advancing to PRT with hand-held weights from approximately POD 53 as tolerated^12)^. By POD 53, the MRCss had improved to 40, handgrip strength to 6.1 kg, the BI to 35, and the EQ-5D-3L to 0.150. By POD 65, short-distance independent ambulation (4 m) was achieved with an MRCss of 46, handgrip strength of 6.9 kg, SPPB 4, usual gait speed of 0.46 m/s, and a BI of 60, whereas longer distances still required assistance around POD 67. In the post-acute facility (POD 74–102), gait and endurance were progressively advanced; at the time of transfer (POD 74), the MRCss was 48, handgrip strength 7.2 kg, SPPB 7, usual gait speed 0.74 m/s, BI 75, and EQ-5D-3L 0.639, and household-level independent ambulation was achieved by approximately POD 83. At discharge (POD 102), the patient showed near-complete functional recovery (MRCss 57; handgrip 15.7 kg; SPPB 12; usual gait speed 1.33 m/s; BI 95) and an EQ-5D-3L of 0.746, exceeding the preoperative value. The timing of key steps is depicted in Figure 1, and the temporal alignment of clinical and functional milestones is summarized in Supplemental Table 1.

### Informed consent and ethical approval

This study was conducted in accordance with the principles of the Declaration of Helsinki and the guidelines of our institution. Written informed consent was obtained from the patient for the publication of this case report. The study protocol was approved by the Kameda Medical Center, Research Ethics Committee (Approval No. Case23-018).

## Discussion

This case illustrates the potential for gradual recovery of a patient with severe ICUAW over an extended period. Preoperative physical function assessments may aid in setting individualized rehabilitation goals and planning referrals to post-acute care facilities.

### Recovery trajectory in severe ICUAW

Recovery from ICUAW varies considerably among individuals, with some patients requiring several months or longer to regain function^4)^. Previous studies have reported that the MRCss at ICU discharge typically ranges from 40 to 50 in patients with ICUAW^7,8)^. However, reports describing the recovery process in patients discharged from the ICU with markedly low MRCss, as in the present case, remain limited. In this case, the patient had a severely reduced MRCss of 16, well below the threshold of 36 used to define severe ICUAW, yet eventually achieved functional independence. This outcome suggests that a diagnosis of severe ICUAW does not necessarily preclude substantial recovery.

Although reports documenting the recovery course of patients with severe ICUAW are limited, one study has described improvement in MRCss from 4 to 60 on POD 107^13)^. However, the MRCss at ICU discharge was approximately 40. Therefore, presenting a detailed recovery trajectory in patients with extremely low MRCss at ICU discharge holds significant clinical value, and we summarize the temporal alignment of clinical and functional milestones in Supplemental Tables 1 and 2. Many studies on ICUAW have focused on risk factors for onset, prevention, and diagnostic approaches, whereas evidence on determinants of recovery and treatment effects remains limited, particularly at the time of hospital discharge and during longer-term follow-up^1–4)^. Prior reviews suggest potential benefits of early mobilization, rehabilitation interventions, and nutritional therapy, although the strength and consistency of evidence vary across these domains^3,14)^. Notably, while early mobilization during ICU stay can improve physical function at hospital discharge, consistent benefits at 6 months have not been demonstrated, and evidence for rehabilitation initiated after ICU discharge remains limited^14)^. In the present case, despite appreciable weight loss and a low MNA-SF score indicating malnutrition, progressive rehabilitation and gradual nutritional support might have contributed to functional recovery. Early, stepwise mobilization may also have attenuated the ICUAW-related disuse cycle and supported neuromuscular recovery.

### Significance of preoperative physical assessment

Jolley et al.^15)^ proposed a staged framework for ICUAW (Stages 0–3) covering diagnosis and management. In Stage 0 (before ICU), assessment of frailty, gait, activities of daily living, usual activities, and employment is recommended.

In our case, the preoperative profile (BI 100, SPPB 12, usual gait speed 1.14 m/s, grip strength 20.9 kg) was interpreted as preserved baseline reserve and was used to set graded rehabilitation goals and to provide an objective basis for referral to post-acute care. More generally, obtaining a detailed functional profile in patients expected to enter the ICU (Stage 0), such as BI, SPPB, gait speed, grip strength, and MRCss when feasible, may help care teams clarify goal setting, estimate recovery potential after the onset of ICUAW, and plan interventions, thereby improving quality of care.

### Changes in patient-perceived health status

In this case, the patient repeatedly expressed anxiety, asking, “Will I really get better?” This highlights the importance of psychological support for patients with ICUAW. The EQ-5D-3L utility score declined from 0.416 preoperatively to −0.166 by POD 27, reflecting the absence of physical recovery at that time. In rehabilitation, it is crucial to establish shared goals with patients and provide regular feedback on progress to help maintain motivation. However, from a psychological care standpoint, a more systematic approach, such as formal assessment and multidisciplinary intervention for post-intensive care syndrome, may have offered additional benefits.

### Limitations

This report describes a single case; therefore, its generalizability is limited. In the present case, pharmacological treatment, including Kampo medicine, was provided along with nutritional support and swallowing therapy. Although recent narrative reviews have highlighted the potential benefits of Kampo medicine in managing sarcopenia and frailty^16)^, the specific contributions of these multidisciplinary interventions to the recovery of the patient were not evaluated and remain unclear. In this case, the independent effect of rehabilitation could not be isolated from other concurrent interventions. Appropriately designed studies are required to establish the effect of rehabilitation.

## Conclusions

Recovery trajectories in patients with ICUAW vary widely. Even when the MRCss is markedly low at ICU discharge, continued rehabilitation may still result in gaining functional independence. Moreover, for patients scheduled for ICU admission, conducting preoperative physical function assessments, including MRCss, may facilitate clear goal setting and enable more tailored interventions, ultimately contributing to improved quality of care.

## Supplementary Materials

Supplemental table 1. Timeline of Clinical Care and Functional Recovery.

Supplemental table 2. Systemic status and ICU management during ICU stay.

## References

[1] Kress JP, Hall JB: ICU-acquired weakness and recovery from critical illness. N Engl J Med. 2014; 370: 1626–1635. 24758618 10.1056/NEJMra1209390

[2] Fan E, Cheek F, et al.: An official American Thoracic Society Clinical Practice guideline: the diagnosis of intensive care unit-acquired weakness in adults. Am J Respir Crit Care Med. 2014; 190: 1437–1446. 25496103 10.1164/rccm.201411-2011ST

[3] Latronico N, Rasulo FA, et al.: Critical illness weakness, polyneuropathy and myopathy: diagnosis, treatment, and long-term outcomes. Crit Care. 2023; 27: 439. 37957759 10.1186/s13054-023-04676-3 PMC10644573

[4] Hermans G, Van den Berghe G: Clinical review: intensive care unit acquired weakness. Crit Care. 2015; 19: 274. 26242743 10.1186/s13054-015-0993-7 PMC4526175

[5] Le Stang V, Latronico N, et al.: Critical illness-associated limb and diaphragmatic weakness. Curr Opin Crit Care. 2024; 30: 121–130. 38441088 10.1097/MCC.0000000000001135 PMC10919276

[6] Fossat G, Baudin F, et al.: Effect of in-bed leg cycling and electrical stimulation of the quadriceps on global muscle strength in critically ill adults: a randomized clinical trial. JAMA. 2018; 320: 368–378. 30043066 10.1001/jama.2018.9592 PMC6583091

[7] Sidiras G, Patsaki I, et al.: Long term follow-up of quality of life and functional ability in patients with ICU acquired weakness - a post hoc analysis. J Crit Care. 2019; 53: 223–230. 31277049 10.1016/j.jcrc.2019.06.022

[8] Koch S, Wollersheim T, et al.: Long-term recovery in critical illness myopathy is complete, contrary to polyneuropathy. Muscle Nerve. 2014; 50: 431–436. 24415656 10.1002/mus.24175

[9] Tsuchiya A, Ikeda S, et al.: Estimating an EQ-5D population value set: the case of Japan. Health Econ. 2002; 11: 341–353. 12007165 10.1002/hec.673

[10] Shiroiwa T, Fukuda T, et al.: Japanese population norms for preference-based measures: EQ-5D-3L, EQ-5D-5L, and SF-6D. Qual Life Res. 2016; 25: 707–719. 26303761 10.1007/s11136-015-1108-2 PMC4759213

[11] Makita S, Yasu T, et al.: JCS/JACR 2021 Guideline on rehabilitation in patients with cardiovascular disease. Circ J. 2022; 87: 155–235. 36503954 10.1253/circj.CJ-22-0234

[12] Coleman SA, Cunningham CJ, et al.: Progressive resistance training in a post-acute, older, inpatient setting: a randomised controlled feasibility study. J Frailty Sarcopenia Falls. 2021; 6: 14–24. 33817447 10.22540/JFSF-06-014 PMC8017350

[13] Morimoto Y, Sekino M, et al.: Recovery of muscle weakness and physical function in a patient with severe ICU-acquired weakness following pulmonary embolism: a case report. Clin Case Rep. 2018; 6: 1214–1218. 29988636 10.1002/ccr3.1576 PMC6028372

[14] Boelens YFN, Melchers M, et al.: Poor physical recovery after critical illness: incidence, features, risk factors, pathophysiology, and evidence-based therapies. Curr Opin Crit Care. 2022; 28: 409–416. 35796071 10.1097/MCC.0000000000000955 PMC9594146

[15] Jolley SE, Bunnell AE, et al.: ICU-Acquired Weakness. Chest. 2016; 150: 1129–1140. 27063347 10.1016/j.chest.2016.03.045 PMC5103015

[16] Lee HG, Arai I, et al.: A herbal prescription of insamyangyeongtang as a therapeutic agent for frailty in elderly: a narrative review. Nutrients. 2024; 16: 721. 38474849 10.3390/nu16050721 PMC10934365

