# Supplementary material for: Recovery of Physical Function in a Patient with Intensive Care Unit-acquired Weakness after Valvular Surgery: A Case Report
Source: Phys Ther Res. 2026 Feb 12;29(1):66–70. doi: 10.1298/ptr.25-E10366 (PMC13143137; doi:10.1298/ptr.25-E10366)
Supplement: Supplementary file 1 — Supplemental table 1. Timeline of Clinical Care and Functional Recovery. [file ptr-29-66-s001.pdf]

Supplemental table 1. Timeline of Clinical Care and Functional Recovery

POD / Setting	Key clinical management	Rehabilitation milestones	Functional status	HRQOL (EQ-5D-3L)	Nutrition	Weight / BMI
pre-op (General ward)	—	physical assessment	hand-grip 20.9kg; gait speed 1.14 m/s; SPPB 12; BI 100	EQ-5D-3L 0.416	MNA-SF 13	51.0 kg (BMI 22.1)
1–9 (ICU)	MV; VV-ECMO; antibiotics	Postural drainage; passive ROM	—	—	enteral nutrition started; MNA-SF 6	—
10–21 (ICU)	MV until POD 21; antibiotics	initial mobilization; edge-of-bed sitting	MRCss 10-16; BI 0	—	MNA-SF 4	—
21–27 (HCU)	HFNC → LFNC; antibiotics	Transfers; assisted sitting; supported standing	MRCss 16-23; BI 0	EQ-5D-3L −0.166	PICC until POD 37; TPN initiated	52.1 kg (BMI 22.6)
27–41 (General ward)	LFNC until POD 40	Standing/marching	MRCss 23-29; BI 0-20	—	SLT: oral feasible; NST engaged; enteral nutrition until POD 38; oral intake stabilizing	50.0 kg (BMI 21.6)
41–53 (General ward)	Kampo engaged	Gait training started (walker / total assist)	MRCss 29-40; hand-grip 0-6.1 kg; BI 20-35	EQ-5D-3L 0.150	MNA-SF 8	46.5 kg (BMI 20.1)
54–74 (General ward)	—	PRT introduced; Short-distance independent ambulation; longer-distance still assisted	MRCss 46; hand-grip 6.1-6.9 kg; gait speed 0.46 m/s; SPPB 4; BI 35-75	—	MNA-SF 10	44.2 kg (BMI 19.1)
74–101 (post-acute)	—	Independent household ambulation	MRCss 48-57; hand-grip 7.2 kg; gait speed 0.74 m/s; SPPB 7; BI 75-90	EQ-5D-3L 0.639	—	44.9 kg (BMI 19.4)
102 (discharge)	—	Community-level independent ambulation; Stair climbing independence	MRCss 57; hand-grip 15.7 kg; gait speed 1.33 m/s; SPPB 12; BI 95	EQ-5D-3L 0.746	—	45.4 kg (BMI 19.7)

VV-ECMO, veno-venous extracorporeal membrane oxygenation; ROM, range of motion; HFNC/LFNC, high-/low-flow nasal cannula; HCU, high-care unit; PICC, peripherally inserted central catheter; POD, postoperative day; TPN, total parenteral nutrition; SLT, speech-language therapist; NST, nutrition support team; BI, Barthel Index; SPPB, Short Physical Performance Battery; MRCss, Medical Research Council sum score; MV, mechanical ventilation.
