# Supplementary material for: Recovery of Physical Function in a Patient with Intensive Care Unit-acquired Weakness after Valvular Surgery: A Case Report
Source: Phys Ther Res. 2026 Feb 12;29(1):66–70. doi: 10.1298/ptr.25-E10366 (PMC13143137; doi:10.1298/ptr.25-E10366)
Supplement: Supplementary file 2 — Supplemental table 2. Systemic status and ICU management during ICU stay. [file ptr-29-66-s002.pdf]

Supplemental table 2. Systemic status and ICU management during ICU stay.

POD	Key clinical management	RASS	Sedation	Analgesia	CRP (mg/dL)	Pain score	Vasoactive agents	Drains	SOFA score
0	MV	−5	Propofol 60 mg/h	IVPCA Fentanyl 20 μg/h	1.6	−	Dobutamine 4.90 μg/kg/min	Pericardial, mediastinal, and subcutaneous drains in place	12
5	MV; VV-ECMO	−3	Midazolam 2 mg/h	IVPCA Fentanyl 20 μg/h	22.4	CPOT 2	Epinephrine 0.02 μg/kg/min; Norepinephrine 0.06 μg/kg/min; Dobutamine 3.92 μg/kg/min	Drains in place	13
10	MV; initial mobilization	−3 to−2	Dexmedetomidine 32 μg/h	IVPCA Fentanyl 20 μg/h	18.8	CPOT 0	Discontinued	Removed	8
15	MV	−1 to+1	Discontinued	Acetaminophen 2000 mg/day	2.4	CPOT 0 to 1	−	−	8
20	MV	0	−	Discontinued	3.7	CPOT 1	−	−	6

POD, postoperative day; MV, mechanical ventilation; VV-ECMO, veno-venous extracorporeal membrane oxygenation; RASS, Richmond Agitation-Sedation Scale; IV-PCA, intravenous patient-controlled analgesia; CRP, C-reactive protein; CPOT, Critical-Care Pain Observation Tool; SOFA, Sequential Organ Failure Assessment
